# Stem cell culture and differentiation in microfluidic devices toward organ-on-a-chip

**DOI:** 10.4155/fsoa-2016-0091

**Published:** 2017-05-08

**Authors:** Jie Zhang, Xiaofeng Wei, Rui Zeng, Feng Xu, XiuJun Li

**Affiliations:** 1 Department of Chemistry, University of Texas at El Paso, 500 West University Avenue, El Paso, TX 79968, USA; 2 Bioinspired Engineering & Biomechanics Center (BEBC), Xi’an Jiaotong University, Xian 710049, PR China; 3 Biomedical Engineering, University of Texas at El Paso, 500 West University Avenue, El Paso, TX 79968, USA; 4 Border Biomedical Research Center, University of Texas at El Paso, 500 West University Avenue, El Paso, TX 79968, USA

**Keywords:** microfluidic devices, organ-on-a-chip, stem cell, stem cell culture, stem cell differentiation

## Abstract

Microfluidic lab-on-a-chip provides a new platform with unique advantages to mimic complex physiological microenvironments *in vivo* and has been increasingly exploited to stem cell research. In this review, we highlight recent advances of microfluidic devices for stem cell culture and differentiation toward the development of organ-on-a-chip, especially with an emphasis on vital innovations within the last 2 years. Various aspects for improving on-chip stem-cell culture and differentiation, particularly toward organ-on-a-chip, are discussed, along with microenvironment control, surface modification, extracellular scaffolds, high throughput and stimuli. The combination of microfluidic technologies and stem cells hold great potential toward versatile systems of ‘organ-on-a-chip’ as desired.

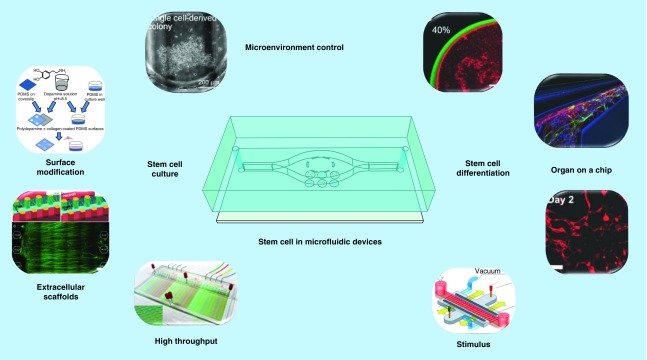

Adapted with permission from [1–8].

## Lay abstract

Stem cells, capable of self-renewing and differentiating into cells of various tissue types, are drawing more and more attention for their enormous potential in many clinically associated applications that include drug screening, disease modeling and regenerative medicine. Conventional cell culture methods, however, have proven to be difficult to mimic *in vivo* like microenvironments and to provide a number of well-controlled stimuli that are critical for stem cell culture and differentiation. In contrast, microfluidic devices offer new capacities and unique advantages to mimic complex physiological microenvironments *in vivo*, and has been increasingly applied to stem cell research.

Stem cells are one kind of cells that have the capability of continuous self-renewal through replication and potential differentiation into specific tissue types [9]. Since the discovery by McCulloch and co-workers in 1963 [10], stem cells have drawn more and more attention for their significant roles in tissue engineering, organ regeneration, cell-based therapies, disease models, drug development and a variety of healthcare applications [11]. Stem cells have been successfully used in healing damaged tissues and replacing nonfunctional organs, etc. Generally, there are two broad types of stem cells: the first type being embryonic stem cells (ESCs), which are isolated from the inner cell mass of blastocysts and have the pluripotency to differentiate into virtually all cell lineages; and the second type being adult stem cells, which are found in various tissues and can differentiate to a limited number of cell types [12]. In addition, the discovery of reprogrammed human-induced pluripotent stem cells (hiPSCs) greatly expanded the realm of stem cell-based research because hiPSCs could side step some ethical issues associated with using human embryonic cells. But the ethical issues are still complex, as discussed in some recent review articles [13,14].

Despite the huge potential of stem cells in many biological and therapeutic areas, major challenges associated with culturing stem cells *in vitro* exist, and some of the challenges include controlled proliferation while maintaining undifferentiated pluripotency and the capability to direct stem cell differentiation reliably [15]. With conventional cell culture methods such as Petri dishes or transwells, however, it is difficult to fulfill these requirements and achieve an *in vivo* like microenvironment in which a variety of well-controlled stimuli are provided for culturing highly sensitive stem cells due to their large-scale and limited reproducibility and reliability [16].

The emerging and rapid development of microfluidic technology has presented an ideal solution for the problem of mimicking an *in vivo* like microenvironment. Microfluidic devices employ precise manipulation of micrometer-to-millimeter-scale fluid flows to achieve high-resolution spatial and temporal controls of the microenvironment [17–22], providing powerful tools for stem cell culture and regulation [23]. Microfluidic platforms are capable of precise manipulation of the microenvironment to deliver soluble factors to cells, construct well-defined gradients, integrate various biocompatible scaffolds and functional components, as well as dynamically alter the application of mechanical signals to cultured cells [24,25]. Tremendous advances have been achieved through combining microfluidic technology with different analysis methods and integrating various structures and functions. Now this technology is widely used in numerous areas such as cell capture and culture, disease diagnosis, single cell analysis, drug screening, metabonomics, proteomics, tissue engineering and other biological applications [26–31]. The combination of microfluidic technologies with stem cell analysis may fill the gap between the present knowledge about stem cells and the in-depth understanding of stem cell mechanisms for their broad practical applications [32–34]. Now there are more and more research efforts focused on the application of microfluidic devices for stem cell research such as stem cell sorting, patterning, culture, differentiation, tissue engineering, organ reconstruction and clinical therapies. Particularly, the concept of organ-on-a-chip, a microfluidic cell culture platform containing continuously perfused chambers with living cells arranged to simulate tissue or organ level physiology, is becoming more and more popular [35]. Advances of microfluidic technologies make it possible to establish an organ model on a microchip, as well as multiple-organ systems by networking different organ models, while stem-cell-derived specific organ cells could be excellent substitutes for human primary cells. The combination of microfluidic technologies and stem cells hold great potential toward versatile systems of organ-on-a-chip as desired. Some other papers have reviewed the significant role of microfluidic devices in stem cell analysis and research from different perspectives [15–16,34,36].

Herein, with this review we will highlight the most recent advances of microfluidic devices for stem cell culture and maintenance, and differentiation toward applications for organ-on-a-chip, particularly with an emphasis on important innovations of different microfluidic aspects to improve stem cell culture and differentiation within the recent 2 years. At the end, the potential of microfluidics to further improve stem cell science and engineering will also be briefly discussed.

## Stem cell culture & maintenance

Stem cells are capable of continued self-renewal and becoming precursor cells of certain specific tissue types. However, stem cells are highly sensitive to various physicochemical cues, and their fate is easily altered by environment changes or loss of the pluripotency; so it is important and challenging to maintain the undifferentiated status of stem cells for further use. A number of stem cell research efforts are concerned with the *in vitro* construction of physiologically relevant cell cultivation environments. Stem cell culture and differentiation require precise control of multiple cues in the cell culture microenvironment [16], which regulate intracellular signaling and ultimately cell phenotype, while it's difficult for conventional culture systems to provide such an accurate control. In this regard microfluidic devices are ideally suited for stem cell culture and maintenance by providing the means to create an *in vivo like* microenvironment, well-defined surface features, patterned scaffolds and substrates, as well as high throughput, as summarized in Table 1.

**Table 1.** **Summary of recent stem cell culture works in microfluidic devices.**

**Features**	**Stem cell type**	**Remarks**	**Ref.**
Microenvironment control for stem cell culture	hiPSCs	Perfusion culture increased the growth rate of hiPSCs	[37]
	hiPSCs	Control shear stress on stem cells	[1]
	hNSCs	Low oxygen and 3D extracellular matrices	[38]
	mESCs	Membrane separated co-culture of mESCs and mEFs	[39]
	hMSCs	Enhanced cryopreservation of MSC monolayer in microfluidic channels	[40]
Surface modification	hBMMSCs	PDMS substrates with varying hydrophobicity, stiffness and roughness	[41]
	hMSCs	Polydopamine coating on PDMS	[2]
	mHSCs	SCF covalently immobilized within GelMA	[42]
	Porcine MSCs	Immobilize collagen type 1 on PDMS	[43]
	hMSCs	Single hMSC differentiation on PAAc	[44]
Scaffolds	hADMSCs, hBMMSCs	MAP gels	[45]
	mNSCs	Alignment in ECM components in 3D hydrogels	[3]
	hASCs, hTMSCs	dECM bioink for bioprinting of cell-laden constructs	[46]
	hMSCs	Hydrogel microbeads based on telechelic POx cross-linkers and the methacrylate monomers (HEMA: METAC: SPMA)	[47]
	mESCs	Core–shell structure to mimic prehatching embryos	[48]
High throughput	hBMMSCs	hBMMSC condensation and 3D micromass culture	[49]
	hBMMSCs	625 microcavities for co-culture of hBMMSCs and HPCs	[50]
	hHSCs	800 chambers to monitor single hHSCs	[51]
	hPSCs	8100 culture chambers for combinatorial screening of bio-factors	[4]

dECM: Decellularized extracellular matrix; ECM: Extracellular matrix; GelMA: Methacrylamide-functionalized gelatin; hADMSC: Human adipose-derived mesenchymal stem cell; hASC: Human adipose-derived stem cell; hBMMSC: Human bone-marrow-derived mesenchymal stem cell; HEMA: 2-hydroxyethyl methacrylate; hHSC: Human hematopoietic stem cell; hiPSC: Human-induced pluripotent stem cell; hMSC: Human mesenchymal stem cell; hNSC: Human neural stem cell; HPC: Hematopoietic progenitor cell; hPSC: Human pluripotent stem cell; hTMSC: Human inferior turbinate-tissue-derived mesenchymal stromal cell; MAP: Microporous annealed particle; mEF: Mouse embryonic fibroblast; mESC: Mouse embryonic stem cell; METAC: [2-(methacryloyloxy)ethyl]trimethylammonium chloride; mHSC: Murine hematopoietic stem cell; MSC: Mesenchymal stem cell; mNSC: Mouse neural stem cell; PAAc: Poly(acrylic acid); PDMS: Polydimethylsiloxane; POx: Poly(2-oxazoline); SCF: Stem cell factor; SPAM: sulfopropyl methacrylate.

### Microenvironment control for stem cell culture

**Figure 1.**
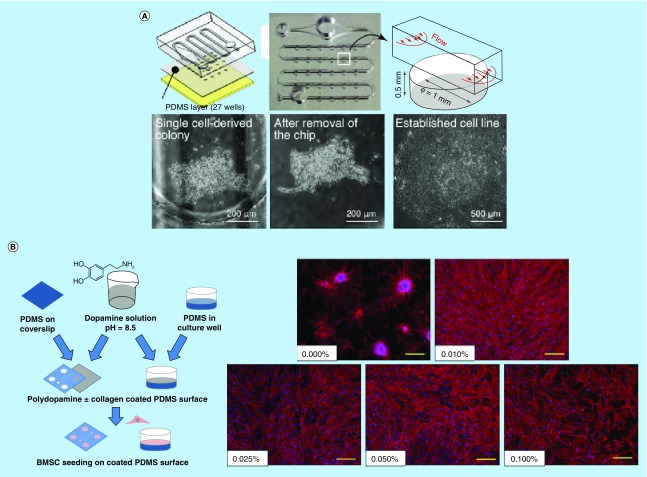

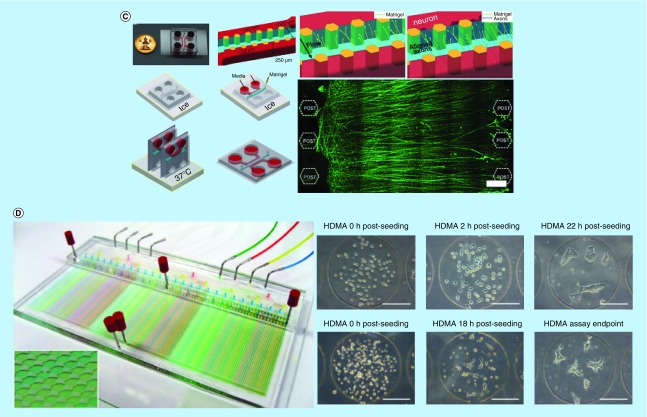
**Stem cell culture in microfluidic platforms.** **(A)** Precise control of shear stress on a single stem cell in a microfluidic device, which established a clonality validated stem cell line after tracing its growth at the single cell level. Reproduced with permission from [1] © Elsevier (2014). **(B)** Polydopamine coating on PDMS for stabilized MSC adhesion and multipotency. Reproduced with permission from [2] © Nature Publishing Group (2015). **(C)** Imparting alignment in ECM components in 3D hydrogels to orient outgrowth of neuronal processes. Reproduced with permission from [3] © Nature Publishing Group (2015). **(D)** A high-throughput microfluidic array containing 8100 culture chambers for hPSC culture and screening of candidate biologicals. Reproduced with permission from [4] © Nature Publishing Group (2016). ECM: Extracellular matrix; HDMA: High-density microbioreactor array; hPSC: Human pluripotent stem cell; MSC: Mesenchymal stem cell; PDMS: Polydimethylsiloxane.

The status of stem cells is highly regulated by their microenvironment, and microfluidic technology has the ability to reconstruct the complex physiological environment suitable for stem cells. As stem cells are highly sensitive to the physicochemical microenvironment, gaining an understanding of the interactions between stem cells and their microenvironments is essential for advancing stem cell research and applications. By controlling the fluidic properties such as convection, diffusion and reaction, microfluidics can tune the microenvironment around stem cells in a variety of ways. For example, Yoshimitsu *et al.* [37] developed a microfluidic perfusion culture system for hiPSCs on a microchamber array chip. Under pressure-driven perfusion culture conditions provided by the microfluidic chip, the growth rate of hiPSCs was found higher than that under static culture conditions. The dynamic microenvironment showed advantages over conventional methods. Another microfluidic culture device developed by Matsumura *et al.* [1] could stably and precisely control the flow of culture medium in channels so as to control the applied shear stress on stem cells. Using this platform, they traced the growth of stem cells at the single-cell level (Figure 1A).

Microfluidic devices were also constructed to mimic *in vivo* like environments for stem cell culture and co-culture. Yang *et al.* [38] presented a microfluidic array for quantitative analysis of human neural stem cells (hNSCs) for self-renewal and differentiation. Thanks to the versatility of these microchips, NSC niche conditions including low oxygen and 3D extracellular matrices such as collagen, fibronectin and laminin were effectively reconstituted to form an *in vivo* like microenvironment. Chen *et al.* [39] proposed a microfabricated approach for feeder-separated co-culture of mouse embryonic stem cells (mESCs) with mouse embryonic fibroblasts on a polydimethylsiloxane (PDMS) porous membrane-assembled 3D microdevice, in which mESC colonies were formed and kept in an excellent undifferentiated state. Due to the support of essential microenvironment factors provided by feeder cells for mESCs; it was found that the purity of mESCs increased due to the separation by the porous membrane. Bissoyi *et al.* [40] reported an interesting work about enhanced cryopreservation of MSC monolayers in microfluidic channels by regulated shear flow. Low shear stress enhanced cell–substrate interaction, cell viability and, subsequently, recovery of adherent human mesenchymal stem cells (hMSCs). The stemness, differentiation potential and adhesion ability of recovered MSCs after cryopreservation were successfully kept with low shear stress treatment, which is critical for preservation of cell monolayer or engineered tissue constructs. These aforementioned reports showed the capability and importance of microfluidic devices in microenvironment control for stem cell culture.

### Surface modification

Surface properties play a significant role in regulating stem cell behaviors including adhesion, proliferation, migration and differentiation. Modification of physicochemical surface properties can be exploited to enhance stable and long-term cell attachment, facilitate efficient cell–substrate interaction and help maintain multipotency of stem cells [52]. Microfluidic technologies enable precise control of reactions and patterning to facilitate surface modifications and engineering in microfluidic devices for stem cells. Menon *et al.* [41] reported a microfluidic assay to induce migration of human bone-marrow-derived mesenchymal stem cells (hBMMSCs) on PDMS substrates with varying combinatorial properties (hydrophobicity, stiffness and roughness), which were easily achieved in a microfluidic device. It turned out that cell proliferation and migration were enhanced on PDMS substrates exhibiting intermediate levels of hydrophobicity, stiffness and roughness. Chuah *et al.* [2] developed a one-step bioinspired polydopamine (PDA) coating strategy to stabilize long-term bone marrow stromal cell culture on PDMS substrates of the microchip (Figure 1B). Changes in surface wettability and the presence of hydroxyl and secondary amines occurred on PDA-coated PDMS surfaces, which contributed to the stability of MSC adhesion, proliferation and multipotency. This simple treatment significantly enhanced the biocompatibility of PDMS-based microfluidic devices for long-term stem cell analysis.

Covalent surface modifications are commonly used to improve the surface properties for stem cell culture. The recent work by Mahadik *et al.* [42] presented a photochemistry-based approach to covalently immobilize stem cell factor (SCF) within methacrylamide-functionalized gelatin (GelMA) hydrogels via acrylate-functionalized polyethylene glycol tethers for *in vitro* culture of primary murine hematopoietic stem cells (mHSCs). Gradients of immobilized SCF were conveniently obtained in GelMA hydrogels by the microfluidic approach for locally directing HSC response. HSCs cultured in GelMA hydrogels with covalently immobilized SCF showed improved selectivity for maintaining primitive HSCs, while induced soluble SCF increased proliferation of differentiating hematopoietic cells. In another study [43], (3-aminopropyl) triethoxy silane and cross-linker glutaraldehyde were employed to immobilize collagen type 1 on PDMS. The modified surfaces were highly efficient to support the adhesion of MSCs with no deterioration of their potency. Although the PDMS substrates were used in most work, some other materials like polystyrene, cyclo-olefin copolymer and Teflon were also used to overcome some drawbacks of PDMS in certain situations such as deformation, evaporation, absorption, leaching and hydrophobic recovery [44,53,54]. For example, Song *et al.* [44] explored adipogenic differentiation of single hMSC on poly(acrylic acid) and polystyrene micropatterns, and found that the differentiation was enhanced on the poly(acrylic acid) micropatterns.

### Extracellular scaffolds

With advances in polymer science, various novel functional hydrogels have recently been developed through functionalizing conventional hydrogels for certain special properties to efficiently act as extracellular scaffolds for stem cell culture [55–57]. A recent trend in microfluidic devices is to use hydrogels as more physiologically similar 3D matrix for stem cells. Taking advantage of microchips for precise spatial control, hydrogels containing cells can be molded into different geometries with various guiding structures such as ridges and pillars. Griffin *et al.* [45] demonstrated an injectable, interconnected microporous gel scaffold assembled from annealed microgel building blocks whose chemical and physical properties could be tailored by microfluidic fabrication. *In vitro*, stem cells incorporated during scaffold formation proliferated well and formed extensive 3D networks, while *in vivo* the scaffolds facilitated cell migration that resulted in rapid cutaneous tissue regeneration. Additionally, Jang *et al.* [3] reported a microfluidic approach to impart alignment in extracellular matrix (ECM) components in 3D hydrogels by tilting at 90° to generate continuous fluid flow across the bulk gel during Matrigel gelation, as shown in Figure 1C. About 70% of the ECM components were oriented along the direction of flow, in which primary rat cortical neurons and mNSCs exhibited oriented outgrowth of neuronal processes within the 3D Matrigel matrix. Pati *et al.* [46] utilized novel decellularized ECM bioink for bioprinting of cell-laden constructs, providing an optimized microenvironment conducive to the growth of 3D structured tissue. Tissue printing was performed with decellularized ECM bioink that encompassed either living human adipose-derived stem cells or human inferior turbinate-tissue derived mesenchymal stromal cells and achieved higher levels of cell viability, differential lineage commitment and ECM formation.

In addition, the ease of microdroplet generation provides another microarchitecture of extracellular scaffolds for stem cell studies. Lück *et al.* [47] presented the synthesis of hydrogel microbeads in a microfluidic device based on telechelic poly(2-oxazoline) (POx) cross-linkers and the methacrylate monomers (HEMA: 2-hydroxyethyl methacrylate; METAC: [2-(methacryloyloxy)ethyl]trimethylammonium chloride; SPAM: sulfopropyl methacrylate) by inverse emulsion polymerization. While neutral, hydrophilic POx–PHEMA (poly HEMA) beads were bioinert, and excessive proliferation of hMSCs on charged POx–PMETAC (poly METAC) and POx–PSPMA (poly SPMA) was observed. Additional collagen I coating further improved the stem cell proliferation. Another novel core–shell microcapsule system was developed by Agarwal *et al.* [48] to mimic the miniaturized 3D architecture of prehatching embryos with an aqueous liquid-like core of embryonic cells and a hydrogel shell of zona pellucida. The cell amount could be precisely controlled in each droplet by the microfluidic device. About 20 mESCs in the core could proliferate to form a single ESC aggregate in each microcapsule within 7 days. Quantitative real-time (RT)-PCR analyses show significantly higher expression of pluripotency marker genes in the 3D-aggregated ESCs.

### High throughput

While conventional tissue culture methods require significant amounts of stem cells and reagents for testing under different culture conditions, microfluidics offer a revolutionary way to perform high-throughput culture and analysis by employing multiple cell cultivation chambers for multiplexed stem cell analysis, holding advantages such as requiring much lower cell amounts yet having a much higher screening efficiency. Occhetta *et al.* [49] designed a high-throughput microfluidic platform with 60 cubic culture chambers for hBMMSC condensation and subsequent culture of 3D micromasses of hBMMSCs under continuous flow perfusion with different concentrations of morphogens being delivered to specific culture units based on a serial dilution generator. Wuchter *et al.* [50] established another 3D co-culture system with 625 microcavities based on a 3D-KITChip as an *in vitro* model system of the human HSC niche. Human bone marrow MSCs together with umbilical cord blood hematopoietic progenitor cells were inoculated in the microcavities, and the MSCs grew in several layers and formed a cellular network in which hematopoietic progenitor cells could fully integrate, while higher expression of specific stem cell markers was achieved over standard co-culture conditions. Recently, Cambier *et al.* [51] presented a large array of 800 chambers which allowed the monitoring of single HSCs. The chamber medium can be renewed by diffusion within a few minutes which would allow the staining of live human HSCs with fluorescent primary antibodies to reveal their stage in the hematopoiesis differentiation pathway. Furthermore, to facilitate high-throughput combinatorial screening of candidate biologicals or factors driving relevant molecular pathways, Titmarsh *et al.* [4] developed a high-density microbioreactor array – a microfluidic cell culture array containing 8100 culture chambers (Figure 1D). Human pluripotent stem cells were cultured in this platform for a combinatorial screening of putative proliferation factors in human pluripotent stem cell-derived cardiomyocytes. High-throughput cell culture also provides much more abundant information than ordinary platforms such as cell proliferation, differentiation, molecular secretion, gene and protein expression, collected from hundreds of parallel chambers. Besides, it is crucial to increase the ability of microfluidic systems to monitor more variables and supply more information of different aspects. For example, Super *et al.* [58] developed a microfluidic device for RT monitoring of specific oxygen uptake rates of ESCs. The system was capable of RT monitoring of cell growth from phase contrast microscopy images and respiration from optical sensors for dissolved oxygen.

## Stem cell differentiation

The potential of stem cells to generate various differentiated cell types offers the possibility of establishing preclinical drug screening platforms or disease models and to create cell sources for regenerative medicine. *In vivo* and *in vivo* like microenvironments can enhance the physiological relevance of the information retrieved from such studies [59]. Stem cell therapy has a wide range of applications from treating diseases, such as cancer and diabetes, to cell repair therapies for wound healing following trauma [11]. The primary step in stem cell therapy is to direct the differentiation of the cells to the desired progeny [60]. The development of *in vitro* stem cell differentiation regulation systems is critical to in-depth understanding of stem cell behaviors and mechanisms for the efficient direction of desired stem cell differentiation, thus leading to desired progeny for various applications. The capacity of microfluidic systems to provide defined and reproducible stimulation scenarios opens a new horizon for more reliable investigation of cell behaviors in an environment that mimics a living tissue. Most recent advances of studies on stem cell differentiation in microfluidic devices are summarized in Table 2.

**Table 2.** **Summary of recent stem cell differentiation works in microfluidic devices.**

**Features**	**Stem cell type**	**Remarks**	**Ref.**
Microenvironment control for stem cell differentiation	hiPSCs	Aligned PDMS microgrooves as physical guidance cues for hiPSC neural differentiation	[61]
	mNSC	MF (defined as the volume of stem cell culture medium divided by the total number of cells at seeding and number of hours between medium replacement) relationship with mNSC differentiation	[62]
	mESCs	Heparin hydrogel droplets containing nodal and FGF-2 to direct mESC differentiation	[63]
	hNSCs	Alginate hollow hydrogel spheres internally coated with Matrigel layers for hNSC differentiation	[5]
	mESCs	Simultaneous or sequential orthogonal gradient	[64]
Stimulus	hMSCs hNSCs	Co-cultured of hNSCs with genetically engineered hMSCs overexpressing GDNF for neuronal differentiation	[65]
	hMSCs	Extremely low shear stress enhanced osteogenic differentiation with TAZ as the mediator	[66]
	ADMSCs	Stretchable PDA-coated parafilm providing mechanical, chemical, biological and topographic cues for osteogenic differentiation	[67]
	hPDMCs	Combining chemical stimulation and sheer stress to promote stem cell differentiation	[68]
	hMSCs	Controlled and simultaneous mechanical, electrical and biochemical stimulations	[6]
Tissue engineering	hiPSCs	Differentiation of hiPSC-derived human neuroepithelial cells into functional dopaminergic neurons	[7]
	hiPSCs	Heart-on-chip for modeling BTHS	[69]
	mESCs	Different cell encapsulation in hydrogel microbeads at different ratios	[70]
	hBMMSCs	3D functional, perfusable microvascular networks composed of human endothelial cells and hBMMSCs	[8]
	mHSCs	Bone-marrow-on-a-chip	[71]

ADMSC: Adipose-derived mesenchymal stem cell; BTHS: Cardiomyopathy of Barth syndrome; GDNF: Glial cell-derived neurotrophic factor; hBMMSC: Human bone-marrow-derived mesenchymal stem cell; hiPSC: Human-induced pluripotent stem cell; hMSC: Human mesenchymal stem cell; hNSC: Human neural stem cell; hPDMC: Human placenta-derived multipotent stem cell; mESC: Mouse embryonic stem cell; MF: Medium factor; mHSC: Murine hematopoietic stem cell; mNSC: Mouse neural stem cell; PDA: Polydopamine; PDMS: Polydimethylsiloxane; TAZ: Transcriptional coactivator with PDZ-binding motif.

### Microenvironment control for stem cell differentiation

Appropriate microenvironments not only promote stem cell maintenance, but also regulate the differentiation of stem cells to achieve homeostasis. In microfluidic devices, the microenvironment of stem cells including flow conditions, soluble factors and extracellular matrix can be precisely controlled to direct stem cell differentiation. Hesari *et al.* [61] developed a hybrid microfluidic system to produce a dynamic microenvironment by placing aligned PDMS microgrooves on the surface of biodegradable polymers as physical guidance cues for controlling the neural differentiation of hiPSCs. The expression of neuronal-specific genes was found to be significantly higher on the microfluidic device compared with conventional systems, an indication of enhanced differentiation of hiPSCs to neuronal cells in the microfluidic device. In another study, Wang *et al.* [62] explored the correlation between the availability of cell culture medium and spontaneous neuronal cell differentiation of murine NSCs. A series of microchannels with specific geometric parameters were designed to provide different amounts of culture medium to the cells over time. It was successfully demonstrated that the amount of culture medium was correlated to neuronal cell differentiation, indicating the importance of the microfluidic design criteria in directing stem cell fates.

**Figure 2.**
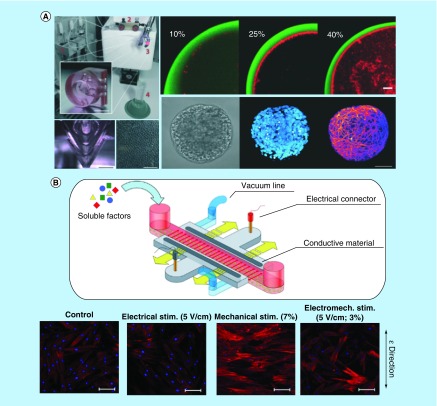
**Stem cell differentiation on microfluidic devices.** **(A)** Core–shell hydrogel droplets for culture and differentiation of hNSCs. Matrigel was coated on the inside surface to mimic the basal membrane. Reproduced with permission from [5] © The Royal Society of Chemistry (2016). **(B)** Combined mechanical, electrical and biochemical stimulations for hMSC differentiation. Reproduced with permission from [6] © the Nature Publishing Group (2015). hMSC: Human mesenchymal stem cell; hNSC: Human neural stem cell.

Microenvironments of gel-based microfluidic systems were also extensively studied to improve cell differentiation. A high-throughput droplet microfluidic platform was developed by Siltanen *et al.* [63] for generating bioactive stem cell-laden microgels to direct stem cell differentiation in a well-defined microenvironment. Mouse ESCs were encapsulated into heparin-containing hydrogel particles with Nodal and FGF-2, which are implicated in specifying pluripotent cells to definitive endoderm, and were found to express high levels of endoderm markers of Sox17 and FoxA2. As shown in Figure 2A, Alessandri *et al.* [5] presented a different gel-based microfluidic device that generates submillimetric alginate hollow hydrogel spheres which were internally coated with a matrigel layer of a few micrometer thick to mimic the basal membrane, and provide a physiologically relevant microenvironment for encapsulating cells. hNSCs derived from hiPSCs were encapsulated and further differentiated into neurons within the capsules with negligible loss of viability. Moreover, Uzel *et al.* [64] described a microfluidic design for generating a diffusion-driven, simultaneous or sequential, orthogonal linear concentration gradients in a 3D-cell-containing scaffold to create a microenvironment of different conditions. Stem cells are subjected to orthogonal gradients of morphogens, and motor neurons preferentially differentiate into regions of high concentration of retinoic acid and smoothened agonist similar to *in vivo* situations.

### Stimulus

As stem cells are sensitive to various environmental cues, investigation of the stem cell differentiation under biochemical and physical stimulations is of great interest. Under traditional Petri dish culturing conditions, it is difficult to control soluble factors precisely or to apply physical stimulation on the stem cells. Microfluidic chips, on the other hand, provide a manageable platform to stimulate cells not only by biochemical cues, but also by structural cues, mechanical stress and electromagnetic forces, among others. Yang *et al.* [65] recapitulated *in vivo* like paracrine signaling of hMSCs in 3D ECMs within a microfluidic array platform to enhance functional neuronal differentiation of hNSCs. Genetically engineered hMSCs, which overexpressed glial cell-derived neurotrophic factor, were co-cultured with hNSCs, leading to reduced glial differentiation of hNSCs and enhanced differentiation into neuronal cells including dopaminergic neurons. Besides, with defined geometries and controlled perfusion flow rates, microfluidic chips provide an *in vitro* cell culture platform that allows precise mimicking of the shear stress in the physiological environment. Kim *et al.* [66] reported increased osteogenic differentiation of hMSCs within an osmotic pump-driven microfluidic chip that generates constant and extremely low shear stress. The low shear stress stimulation significantly induced TAZ (transcriptional coactivator with PDZ-binding motif) nuclear localization and transcriptional activity, thereby facilitating osteogenic differentiation. Shi *et al.* [67] constructed a microfluidic cell culture platform that integrated stretchable PDA-coated parafilm supporting stem cell adhesion and proliferation. Adipose-derived MSCs that were cultured on the PDA-coated parafilm with grooved micropatterns exhibited significantly higher osteogenic commitment in response to mechanical and spatial cues, compared with the cells without stretching.

Occasionally, multiple stimulations were applied for stem cell differentiation in microfluidic devices. For instance, Cheng *et al.* [68] combined chemical stimulation and sheer stress to promote stem cell differentiation. Human placenta-derived multipotent stem cells were successfully cultured on a microfluidic platform and induced to differentiate into neuronal cells by 1-methyl-3-isobutylxanthine stimulation. During this process, different shear forces were applied by adjusting the flow rate of 1-methyl-3-isobutylxanthine solution injection, and was found to accelerate the placenta-derived multipotent stem cells’ differentiation into neural cells. Furthermore, Pavesi *et al.* [6] developed a microscale cell stimulator capable of providing controlled and simultaneous mechanical, electrical and biochemical stimulations, as shown in Figure 2B. Each stimulation could be applied independently or combined to study the interactions of multiple stimuli for more accurate representations of complex *in vivo* situations. Mechanical stimulation was found to induce morphological changes and actin cytoskeletal rearrangements in hMSCs. Changes in gene expression proved that either mechanical or electrical stimulation helped induce activation of cardiac myocyte markers.

### Organ-on-a-chip

**Figure 3.**
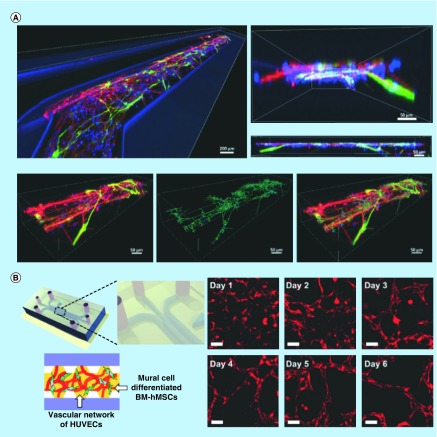
**Stem cell-based organ-on-a-chip construction.** **(A)** Differentiation of hiPSC-derived human neuroepithelial cells into functional dopaminergic neurons in microchannels. Reproduced with permission from [7] © The Royal Society of Chemistry (2015). **(B)** Generation of 3D functional microvascular networks with hMSCs in a microfluidic system. Reproduced with permission from [8] © The Royal Society of Chemistry (2014). hiPSC: Human-induced pluripotent stem cell; hMSC: Human mesenchymal stem cell.

Organ-on-a-chip is based on microfluidic cell culturing to model physiological functions of tissues and organs. Currently, the focus is not to rebuild a whole living organ, but to mimic minimal functional units that recapitulate tissue and organ level functions. Although most related studies have been carried out using established cell lines or primary cells, the use of stem cells is increasing because of the tremendous potential to model various disease models or biological systems. Precise control of stem cell differentiation in the microfluidic microenvironment makes tissue engineering and organ-on-a-chip developments become more promising [72–75]. To date, a number of proof-of-concept, organ-on-a-chip systems using cells differentiated from stem cells have been described [16,24]. As shown in Figure 3A, Moreno *et al.* [7] used hiPSCs to derive human neuroepithelial cells and successfully differentiated them into functional dopaminergic neurons within phase-guided 3D microfluidic cell culture bioreactors. After 30 days of differentiation, *in situ* morphological, immunocytochemical and electrophysiological characterization confirmed the presence of dopaminergic neurons that were spontaneously, electrophysiologically active. Wang *et al.* [69] combined patient-derived and genetically engineered iPSCs with tissue engineering to elucidate the pathophysiology underlying the cardiomyopathy of Barth syndrome (BTHS) through ‘heart-on-chip’. BTHS iPSC-derived cardiomyocytes assembled into sparse and irregular sarcomeres, which contracted weakly similar to *in vivo* situations. Abnormalities with mitochondrial function caused by TAZ mutation and cardiolipin deficiency were identified using this platform, and proved to be necessary and sufficient to disrupt sarcomere assembly and contractile stress generation. Also, droplets technology was commonly used for stem cell niche engineering and organ reconstruction. Tumarkin *et al.* [70] presented a microfluidic platform for high-throughput generation of hydrogel microbeads containing different cell populations in which the cell ratio was controlled by changing the volumetric flow rates of the corresponding streams. Factor-dependent and responsive blood progenitor cell line MBA2 and M07e cells at varying ratios were co-encapsulated and showed that in-bead paracrine secretion can modulate the viability of the factor (IL-3) dependent cells.

Moreover, many tissue modeling efforts were focused on the reconstruction of microvascular networks in microfluidic devices based on stem cells, evidence of the advantages of microfluidics being an appropriate platform for allowing the perfusion vessels and incorporation of the accompanying shear stresses. As shown in Figure 3B, Jeon *et al.* [8] developed a 3D functional and perfusable microvascular network composed of human endothelial cells and hBMMSCs phenotypically transitioning toward mural cells by using a vasculogenesis-like approach. TGF-β1 was found to have an important effect on the hBMMSCs’ phenotypic transition, but not to allow the generation of functional microvascular networks, while angiopoietin supplemented systems formed interconnected and perfusable microvessels. Interestingly, Torisawa *et al.* [71] reported the fabrication of ‘bone-marrow-on-a-chip’ which was composed of artificial bone and living marrow with a functional hematopoietic niche *in vitro*. A hollow compartment was first filled with type I collagen gel and bone-inducing materials. The device was then implanted subcutaneously in a mouse for *in vivo* engineering of bone marrow. After 4–8 weeks, the whole device was removed from the mouse and inserted into a microfluidic device. In this engineered bone marrow, hematopoietic stem and progenitor cells were kept *in vivo* like proportions for at least 1 week.

## Conclusion & future perspective

In conclusion, the field of stem cell research has been significantly advanced by the microfluidic technology. Microfluidic devices, which can control multiple soluble and physical factors simultaneously over space and time with high precision, provide an ideal and well-defined platform for stem cells, which are quite sensitive to the surrounding microenvironment, and enable a better understanding of the biochemical and biophysical regulations of stem cell fates. In this review, we focused on recent advances of microfluidic devices for stem cell culture and maintenance with retained pluripotency, and controlled stem cell differentiation into specific cell types or tissues toward the goal of developing organ-on-a-chip, as summarized in Tables 1 & 2. Various types of stem cells such as ESCs, neural stem cells, induced PSCs, HSCs and MSCs derived from different tissue sources were integrated in different microfluidic systems. Thanks to the ability of microfluidics to control stem cell microenvironments with high spatial and temporal precision and to conduct experiments in conditions resembling *in vivo* situations through properly designed microstructures, surface modification and integration of biocompatible extracellular materials, it is feasible to maintain a suitable microenvironment for stem cell natural growth. Additionally, by making use of parallel microstructures and/or microdroplets, microfluidic platforms are capable of high-throughput stem cell culture and analysis. Furthermore, surpassing conventional methods, microfluidic devices are able to integrate biochemical and physical factors simultaneously to provide defined and reproducible stimulation for precisely controlled differentiation of stem cells, which is critical for stem-cell-based therapies such as wound healing and organ reconstruction. The unique capability of microfluidics over conventional methods has advanced stem cell research. For example, ‘bone-marrow-on-a-chip’ exhibited organ-level marrow toxicity responses and protective effects of radiation countermeasure drugs, while conventional bone marrow culture methods do not [71], and the heart disease model of BTHS on a chip provided new insights into the pathogenesis and potential treatment strategies [69]. In the future, more and more microfluidic devices are expected to be developed and applied for stem cell research, particularly toward the goal of organ-on-a-chip and clinical applications.

In the light of an increasing demand of stem cells for disease modeling, drug screening and cell-based therapies, a large number of highly characterized stem cells and derivatives will be in great demand. Most current microfluidic platforms, however, are custom designed and fabricated for some particular applications and cannot meet the requirement of large-scale commercial applications. Wider implementation of these systems will require greater access of standardized microfluidic systems for the general stem cell research community. In addition, the production of large quantities of specific cells is challenging to carry out in a controlled and well-defined manner using microfluidic systems. The scale-up of microfluidic systems and parallel processes are needed. Besides, limited by dimensions, it is difficult to achieve large tissues and organs within microfluidic channels, which may need novel designs and combination with other technologies such as bioprinting. Although current microfluidic technologies are confronted with some challenges, through the integration of new technologies and materials as well as standardization and automation, we believe that diverse microfluidic devices will be used extensively in stem cell research and will become a powerful tool for both fundamental studies and medical applications of stem cells in the near future.

**Executive summary**

**Background**

- Stem cells have huge potential of biological and medical applications, but conventional methods are difficult to provide *in vivo* like microenvironment, which is crucial for stem cell culture and differentiation.
- With unique advantages, microfluidic lab-on-a-chip provides unprecedented opportunities for stem cell research.

**Stem cell culture & maintaining**

- Stem cells are highly regulated by their microenvironments, and microfluidic technology has the ability to reconstruct complex physiological environments suitable for stem cells through accurate flow control, modification of physiochemical surface properties and the use of patterned hydrogels as physiologically similar to 3D matrixes.
- Microfluidics offer high-throughput culture and analysis by employing multiple cell cultivation chambers for multiplexed stem cell analysis.

**Stem cell differentiation**

- Precise control of dynamic microenvironments and multiple stimuli for stem cell differentiation in microfluidic devices pave the way for tissue engineering and organ-on-a-chip.

**Conclusion**

- Although confronted with some challenges, microfluidic devices are expected to be used extensively in stem cell research in the future, particularly toward the goal of organ-on-a-chip.
